# International veterinary epilepsy task force consensus proposal: outcome of therapeutic interventions in canine and feline epilepsy

**DOI:** 10.1186/s12917-015-0465-y

**Published:** 2015-08-28

**Authors:** Heidrun Potschka, Andrea Fischer, Wolfgang Löscher, Ned Patterson, Sofie Bhatti, Mette Berendt, Luisa De Risio, Robyn Farquhar, Sam Long, Paul Mandigers, Kaspar Matiasek, Karen Muñana, Akos Pakozdy, Jacques Penderis, Simon Platt, Michael Podell, Clare Rusbridge, Veronika Stein, Andrea Tipold, Holger A Volk

**Affiliations:** Department of Pharmacology, Toxicology and Pharmacy, Ludwig-Maximillians-University, Königinstr. 16, 80539 Munich, Germany; Service Neurology at the Centre for Clinical Veterinary Medicine, Ludwig-Maximilians-University, Veterinärstr. 13, 80539 Munich, Germany; Department of Pharmacology, Toxicology and Pharmacy, University of Veterinary Medicine Hannover, Bünteweg 17, 30559 Hannover, Germany; University of Minnesota College of Veterinary Medicine, D426 Veterinary Medical Center, 1352 Boyd Avenue, St. Paul, MN 55108 USA; Department of Small Animal Medicine and Clinical Biology, Faculty of Veterinary Medicine, Ghent University, Salisburylaan 133, Merelbeke, 9820 Belgium; Department of Veterinary and Clinical Sciences, Faculty of Health and Medical Sciences, University of Copenhagen, Frederiksberg C, Denmark; Animal Health Trust, Lanwades Park, Kentford, Newmarket, CB8 7UU Suffolk, UK; Fernside Veterinary Centre, 205 Shenley Road, Borehamwood, SG9 0TH Hertfordshire UK; University of Melbourne, 250 Princes Highway, Weibee, 3015 VIC Australia; Department of Clinical Sciences of Companion Animals, Utrecht University, Yalelaan 108, 3583 CM Utrecht, The Netherlands; Section of Clinical & Comparative Neuropathology, Centre for Clinical Veterinary Medicine, Ludwig-Maximilians-University, Veterinärstr. 13, 80539 Munich, Germany; Department of Clinical Sciences, College of Veterinary Medicine, North Carolina State University, 1052 William Moore Drive, Raleigh, NC 27607 USA; Clinical Unit of Internal Medicine Small Animals, University of Veterinary Medicine, Veterinärplatz 1, 1210 Vienna, Austria; Vet Extra Neurology, Broadleys Veterinary Hospital, Craig Leith Road, Stirling, FK7 7LE Stirlingshire UK; College of Veterinary Medicine, University of Georgia, 501 DW Brooks Drive, Athens, GA 30602 USA; Chicago Veterinary Neurology and Neurosurgery, 3123 N. Clybourn Avenue, Chicago, IL 60618 USA; Fitzpatrick Referrals, Halfway Lane, Eashing, Godalming, GU7 2QQ Surrey, UK; School of Veterinary Medicine, Faculty of Health & Medical Sciences, University of Surrey, Guildford, GU2 7TE Surrey UK; Department of Small Animal Medicine and Surgery, University of Veterinary Medicine Hannover, Bünteweg 9, 30559 Hannover, Germany; Department of Clinical Science and Services, Royal Veterinary College, Hatfield, AL9 7TA Hertfordshire UK

**Keywords:** Dog, Epileptic seizure, Epilepsy, Treatment

## Abstract

Common criteria for the diagnosis of drug resistance and the assessment of outcome are needed urgently as a prerequisite for standardized evaluation and reporting of individual therapeutic responses in canine epilepsy. Thus, we provide a proposal for the definition of drug resistance and partial therapeutic success in canine patients with epilepsy. This consensus statement also suggests a list of factors and aspects of outcome, which should be considered in addition to the impact on seizures. Moreover, these expert recommendations discuss criteria which determine the validity and informative value of a therapeutic trial in an individual patient and also suggest the application of individual outcome criteria. Agreement on common guidelines does not only render a basis for future optimization of individual patient management, but is also a presupposition for the design and implementation of clinical studies with highly standardized inclusion and exclusion criteria. Respective standardization will improve the comparability of findings from different studies and renders an improved basis for multicenter studies. Therefore, this proposal provides an in-depth discussion of the implications of outcome criteria for clinical studies. In particular ethical aspects and the different options for study design and application of individual patient-centered outcome criteria are considered.

## Background

Therapeutic management of canine and feline patients with epilepsy poses a particular challenge for the practitioner. The challenge is related to the multitude of etiologies as well as the high inter-individual variance in the clinical picture of canine and feline epilepsies. Moreover, the response to standard therapeutic regimes differs tremendously between individual patients.

Standardization in the assessment and reporting of outcome of therapeutic interventions is essential for several reasons. In individual patients, standardized procedures in the evaluation of therapeutic responses will guide practitioners in the diagnosis of drug resistance as a basis for the decision to continue with an alternate therapeutic regime. Moreover, expert consensus based recommendations render a basis for common reporting schemes, which can significantly improve the information content of patient history documents e.g. in the case of referral to a veterinary neurology specialist. Thus, one aim of this consensus proposal is to provide expert recommendations for the assessment of outcome in individual patients focusing on the impact on seizures but also considering other relevant aspects of outcome. In addition, we provide and discuss a list of criteria that determine whether a therapeutic trial in an individual patient can be considered adequate and informative. Respective guidelines will also help to exclude pseudo-resistance (defined as lack of a response due to an inadequate dosing or treatment regime) in individual patients.

Standardized assessment and reporting of therapeutic outcome in individual patients also is a prerequisite for the realization of scientifically proven clinical studies. In general, it is of particular relevance for the informative value of the study that strict inclusion and exclusion criteria are considered in the enrolment of patients for clinical studies evaluating a particular therapeutic regime. For instance if the study plan is to enrol patients, in which epilepsy proved to be resistant to monotherapy with a specific antiepileptic drug, a common definition of resistance as well as common criteria for an adequate and informative trial are needed urgently. Thus, universal recommendations provided in this proposal will render a basis for an improved consideration of inclusion and exclusion criteria, will help reduce study population variance, and will thereby increase the significance of study data sets and findings.

Considering the diversity of etiologies and phenotypes of canine and feline epilepsy and considering the fact that data from human patients indicate that therapeutic responses differ tremendously between patient subgroups depending on etiology, epilepsy and seizures types, there is a pressing need to perform clinical studies in respective subgroups of canine and feline patients. Studies focusing on epilepsy with a specific etiology will only be feasible in the form of multicenter studies, which require common schemes for outcome assessment. Thus, one purpose of this consensus paper is to provide the scientific, practical and ethical aspects to be considered in different types of epilepsy study designs.

## Assessment of outcome in individual patients

### Impact on seizures: definition of drug resistance and of therapeutic success in individual patients

Despite a high number of studies dealing with the clinical issue of drug resistance, a common definition of drug resistant epilepsy is lacking. In 2010, a Task Force established by the International League against Epilepsy (ILAE) has proposed a working definition for drug resistance in human patients, which since then has been assessed in clinical practice: “*Drug resistant epilepsy is defined as failure of adequate trials of two tolerated, appropriately chosen and used antiepileptic drug schedules (whether as monotherapies or in combination) to achieve sustained seizure freedom*” [1]. This definition has been the source of much debate in relation to human epilepsy, and is intended mainly for epidemiological work rather than to guide individual practice. A recent study evaluated and confirmed the reliability and validity of the criteria provided by the definition [2]. The question for veterinary neurology is whether this definition is suitable for the specific conditions in clinical veterinary practice and whether it can be applied to classify the outcome in canine and feline patients.

**Fig. 1:**
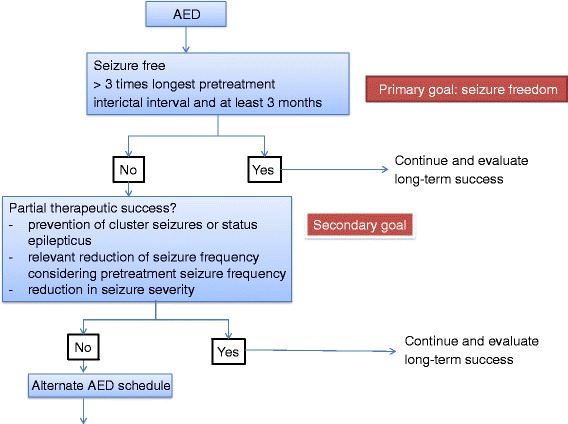
Categorization of seizure control. Seizure freedom is the primary treatment goal in the therapeutic management of canine and feline epilepsy patients. The additional category of partial therapeutic success takes into account that the prevention of seizure clusters or status epilepticus, and a reduction in seizure frequency or seizure severity can be of significant clinical relevance in veterinary patients

There is agreement that *seizure freedom* is the *primary treatment goal* in the therapeutic management of canine and feline epilepsy patients (Fig. 1; Table 1). Striving for complete seizure control is of utmost importance considering the consequences of recurrent seizures. Repeated epileptic seizures can result in neuronal cell loss, persistent neuro-inflammation, disturbance of blood-brain barrier function, and functional alterations in neurotransmitter receptors and ion channels [3–5]. Respective alterations can contribute to the development of behavioral co-morbidities, can contribute to a progressively increasing intrinsic disease severity, and a declining responsiveness to therapeutic interventions [6].

**Table 1:** Categorization of outcome in individual patients

Categories: seizure control
1. Seizure-free
2. Seizures continue with partial therapeutic success (specified: reduction in seizure frequency including information on seizure incidence, seizure severity, or reduction in frequency of seizure clusters and status epilepticus)
3. Seizures continue without partial therapeutic success
4. Undetermined (specify reason)
Categories: tolerability
A. No adverse effects
B. Adverse effects
C. Treatment not tolerated (substantial adverse effects resulting in discontinuation)
D. Undetermined (specify reason)
Consider that short-term and long-term success should be evaluated and should be indicated as discussed in the text. As outlined in the text respective outcome information should always include information about the drug regime. Table modified from [1].

Sudden unexpected death in epilepsy (SUDEP) is a rare event, which however puts the patient at risk with each single seizure event [7, 8]. Although an overall decreased life-span has not been confirmed in a recent study focusing on idiopathic epilepsy [9], several other reports acknowledge a decreased life-span in canine patients with idiopathic and structural (=symptomatic) epilepsy [10–12]. These reports indicate that euthanasia is the major risk factor contributing to a decreased life-span due to uncontrolled seizures [10–12], but sudden unexpected death in epilepsy (SUDEP) and seizure-related falls, injuries, or asphyxiation are also risk factors in the management of canine patients contributing to increased mortality rates [11, 13]. To our knowledge no information is available yet about SUDEP and life expectancy in feline epilepsy patients.

The ILAE Task Force has acknowledged in their proposal that “a therapeutic intervention may lead to a clinically meaningful reduction in seizure frequency (or severity) that stops short of seizure freedom” [1]. In view of the fact that complete vs. incomplete seizure control does not have the same implications and consequences in veterinary patients as it has in human patients due to the socioeconomic impact on daily lifestyles, and that therapeutic decisions have thus to be balanced with costs and adverse effects, we included the category of *partial therapeutic success* as a *secondary treatment goal* in the classification scheme that we suggest in this proposal (see 2.4) (Fig. 1; Table 1). This decision also takes into consideration that in the past AED induced remission for 1–3 years has only been reported in 15 – 24 % of dogs with idiopathic epilepsy in a wide range of studies focusing on different dog breeds with epilepsy of various severity [11, 12, 14].

The additional category of *partial therapeutic success* takes into account that a reduction in seizure frequency, seizure severity, and the prevention of seizure clusters or status epilepticus can be of significant clinical relevance in veterinary patients (Fig. 1; Table 1). Regarding an impact on seizure frequency it is difficult to set a %-based limit for partial success, because the baseline seizure frequency needs to be taken into consideration. Experience of veterinary neurologists suggests that patient caregivers, the owners, often consider less than one seizure in 3 months acceptable [15]. Thus, depending on the pretreatment seizure frequency a reduction of seizure density to a respective seizure interval e. g. one seizure every 3 months can be considered as a relevant effect. In addition, a reduction in seizure severity can result in a clinically meaningful success, if for instance spread of seizure activity e. g. generalization of focal onset seizures is prevented so that seizures remain focal. Moreover, the prevention of seizure clusters or status epilepticus can significantly affect the quality of life of the patient and the pet owner.

Partial therapeutic success can have a significant clinical relevance in canine and feline patients also affecting the owner’s decision for euthanasia. Nevertheless, we propose to apply the ILAE Task Force definition for veterinary patients thereby drug-resistant epilepsy is diagnosed if seizure freedom is not achieved with two therapeutic trials. However, we suggest indicating for each patient in which drug-resistant epilepsy has been diagnosed if there was evidence for a partial therapeutic success as outlined above.

Moreover, consideration should be given to the fact that there might still be reasonable hope to achieve seizure freedom in patients in which several therapeutic trials have failed. Respective evidence has been reported by different groups performing studies in human patients [16–18]. Neligan et al [17] concluded that about half of the patients with apparent drug resistant epilepsy can have relevant improvements in seizure control with further drug changes. Based on these findings they discussed that the proposed ILAE Task Force definition might be too restrictive [17]. Another study indicated that childhood-onset epilepsy might require specific considerations as 51 % of the patients with drug-resistant epilepsy entered 5-year terminal remissions [18]. Despite the lack of respective, comprehensive data sets in veterinary medicine, we feel that it is important to avoid an early classification of drug resistant epilepsy having a negative impact on the clinician’s efforts to continue with therapeutic trials in individual patients. Thus, we suggest that the term *drug resistant is always used along with the specification ‘to which antiepileptic drugs’*, e.g. phenobarbital resistant, imepitoin resistant and/or bromide resistant [19, 20].

The ILAE task force definition lists an ‘appropriately chosen antiepileptic drug schedule’ as a presupposition for outcome conclusions [1]. In human patients knowledge about pathophysiological mechanisms as well as the outcome of clinical studies rendered the basis for treatment guidelines, which list first line antiepileptic drugs, adjunctive antiepileptic drugs, second line antiepileptic drugs, and antiepileptic drugs that may worsen seizures for different seizure types and epilepsy syndromes [21, 22]. Unfortunately, there is a lack of knowledge about drug responsiveness of different seizure types and of epilepsies with different etiologies in veterinary medicine. Despite this fact we propose to keep the term ‘appropriately chosen’ in the definition (see consensus statement on treatment for recommendations [23]), when applying it to veterinary patients, as we expect a gain in knowledge in the near future and as it should also motivate to study differential responsiveness in patient subgroups and canine vs. feline patients in more detail.

Please note that criteria for an adequate and informative trial in individual veterinary patients are discussed under 2.4.

### Other criteria and aspects of outcome

#### Impact on neurobehavioral comorbidities

Experimental studies as well as studies in human patients point to a bidirectional link between epileptic seizures and psychological symptoms [24]. In human epilepsy patients the increased prevalence of psychiatric disorders including attention deficit hyperactivity disorder, depression, and anxiety disorders has been attributed to the psychosocial burden of epilepsy but also to epilepsy-associated molecular, cellular, and network alterations. Also, it is postulated that in some instances, the epilepsy and the co-morbidities are both the result of similar underlying mechanisms. A direct impact of pathophysiological mechanisms of epilepsy on neurobehavioral comorbidities is further confirmed by findings in animal models [25]. So far only limited information is available about epilepsy-associated neurobehavioral alterations in veterinary medicine. In drug-naïve dogs diagnosed with idiopathic epilepsy the development of the disease resulted in an increase in the behavioral scores for Fear/Anxiety, Defensive Aggression, and Abnormal Perception [26]. Following onset of medication Defensive Aggression was attenuated, whereas other behavioral alterations became evident including Abnormal Reactivity, Attachment Disorder, Demented Behavior, and Apathetic Behavior [26]. These data underline the need to evaluate the effect of a therapeutic regime on patient’s behavior with a particular focus on a beneficial impact on neurobehavioral comorbidities. Therefore, it is necessary to further develop behavioral scoring systems validated for the assessment of *epilepsy-specific behavioral comorbidities*. The efforts by Shihab et al [26] and Wessmann et al [27] render an important basis for respective score sheets, which are needed urgently for different types of epilepsy in canine and feline patients. In this context the questionnaire developed for the analysis of behavior in Cavalier King Charles Spaniels with neuropathic pain due to Chiari-like malformation should be considered as an example questionnaire tailored for a specific neurological disease [28].

It is emphasized that data need to be collected before the initiation of therapy, because only this baseline information will allow distinguishing between disease-associated alterations as well as beneficial or detrimental effects of antiepileptic drugs. Moreover, despite the fact that controversial findings exist, it is recommended to thoroughly investigate the endocrine status in particular considering that thyroid function might be altered in association with epilepsy development and antiepileptic drug treatment, and that the functional state of the thyroid gland has a major impact on neurobehavior and brain function [29–32].

#### Adverse effects

Tolerability issues constitute an important limiting factor in the therapeutic management of epilepsy in human and veterinary patients [27, 33, 34]. As further discussed below they can significantly contribute to the patient’s burden and can thereby determine drug retention rates. Thus, the extent and the course of adverse effects should be closely monitored when assessing the overall outcome of a therapeutic trial in an individual patient (Table 1). In general, it is important to distinguish between dose-related effects and idiosyncratic effects as well as between transient and long-term effects. Repeated evaluation of adverse effects is necessary during titration phases but also during chronic therapy. It needs to be considered that adjustment and selective tolerance to specific adverse effects can occur, and that aging or development of multi-morbidities might alter the predisposition of individual patients.

Pharmacological targeting of central nervous system hyperexcitability is of course prone to be associated with central nervous system adverse effects. However, pronounced inter-individual differences exist in the susceptibility to respective effects. Sedation or apathy and other behavioral alterations as well as a disturbance of motor function [35, 36], sleep patterns, and cognition [37] are among the dose-dependent central nervous system effects, which should be considered in a patient’s evaluation. In addition, systemic effects need to be assessed including gastrointestinal effects. Moreover, it is well known that the exposure to specific antiepileptic drugs can increase the risk to develop pancreatitis [38, 39], hepatopathy, blood dyscrasias [40, 41] and skin reactions. Specific attention to the following introduction of a new antiepileptic drug should be drawn to the potential development of antiepileptic drug hypersensitivity syndrome [42–45] which may evolve into a life-threatening situation and requires immediate modification of the drug regimen.

Food intake, water intake, body weight gain or loss can be affected by both, central and peripheral effects of antiepileptic drugs. The introduction of standardized validated questionnaires based on Likert or VAS scores which comprise a respective list of frequent and rare adverse effects allowing repeated comparison during drug treatment are highly recommended. Comparison with the pre-drug baseline condition, data, and antiepileptic drug levels is of particular relevance. Evaluation should also include pre-drug baseline and post-drug laboratory evaluation which should ideally include CBC, extended biochemical serum profile, urine and adequate evaluation of liver function (pre- and postprandial bile acids or ammonia). Evaluation of thyroid function is also recommended but faces specific challenges.

In case of polytherapy, putative drug interactions require specific considerations when assessing the tolerability of an antiepileptic drug regime. Despite a controversial discussion, we recommend that the endocrine status is carefully controlled as thyroid function might be affected by the disease as well as its treatment, and might in turn affect the general condition with a pronounced impact on behavior as well as body weight.

In case of severe adverse effects resulting in discontinuation of a specific therapeutic approach, this fact should be documented in the patient’s files with the classification *‘treatment-not-tolerated’* with information about the specific drug or other approach tested e.g. *‘Phenobarbital not tolerated’*.

### Assessment of the impact on quality of life

The impact of a treatment regime on quality of life (QoL) must be considered as a major factor for the evaluation of outcome. Thereby, therapeutic management can affect QoL in a dichotomous manner. Whereas improved seizure control can exert beneficial effects on QoL, adverse effects can contribute to the patient’s burden.

The World Health Organization (WHO) has defined QoL as the individual’s perception of their position in life in the context of the culture and value systems in which they live and in relation to their goals, expectations, standards and concerns [46]. The International Society for QoL Research considers health-related QoL as the functional effect of a medical condition and/or its consequent therapy upon a patient (http://www.isoqol.org). They emphasize that health-related QoL is subjective and multidimensional, encompassing physical and occupational function, psychological state, social interaction and somatic sensation. It is a matter of course that the assessment of health-related quality of life in veterinary medicine is limited to just some selected dimensions and aspects from the list of those considered in human medicine.

Whereas the development of standardized tools can render a basis for patient-reported outcomes measurement in human patients, assessment of the QoL of veterinary patients poses an even greater challenge to veterinary practitioners regardless of the indication. On the other hand it is well known that the perception of a veterinary patient’s QoL by the owner plays a major role in important decisions regarding the therapeutic management of epilepsy or the decision for euthanasia of a patient with difficult-to-treat or drug-resistant epilepsy.

Problems are associated with the fact that the owner’s QoL can constitute a bias in the owner-based evaluation of the QoL of veterinary patients with epilepsy. In this context, it needs to be considered that caring for a dog with idiopathic epilepsy proved to have a major impact on the carer’s QoL [10, 27]. Thus, it is of particular relevance to not only assess the patient’s QoL with owner-based questionnaires but also to assess the carer’s QoL, and consider both in interpretations. In this context, it is of interest that the owner’s perception of their dog’s quality of life proved to negatively correlate with the amount of work required to care for the dog [47].

QoL evaluation should ideally be performed before treatment onset, following treatment initiation, following treatment adjustments regarding dose titration or drug choice, and should be repeated on an annual basis. Whereas patient-related questions in the questionnaire developed by Wessmann et al [27] focused on the control of seizures and adverse effects of antiepileptic drugs, owner-related key questions dealt with restrictions on the carer’s life, frustrations of the carer, the owner’s distaste of antiepileptic drug adverse effects, the carer’s anxiety around the seizure event, and the perception of rectal diazepam use. The efforts by Wessmann et al [27] rendered a validated tool specific for canine idiopathic epilepsy. Muñana et al [48] have applied a QoL assessment in the evaluation of adjunctive levetiracetam efficacy and tolerability. The questionnaire used in this study was adapted from one previously described by Lord and Podell [47]. Respective standardized QoL assessment tools need to be evaluated and if necessary further specified for symptomatic epilepsies, and need to be developed for feline patients.

### Adequate and informative therapeutic trial – criteria

In order to allow valid conclusions about the individual outcome, each therapeutic trial should have been used at optimal doses to exclude pseudoresistance defined as the lack of a response due to an inadequate dosing or treatment regime. As in human medicine, pseudoresistance can have multiple reasons in veterinary patients. First of all the compliance of the patient’s owner should be considered and if in doubt should be controlled by plasma concentration analysis. As also emphasized by Kwan et al [1] for human patients, it is of particular relevance to guarantee an adequate dosing with sufficient duration including efforts for optimization of dosing and titration to clinically efficacious and still tolerated doses. If relevant based on the mechanism of action of an antiepileptic drug, it is recommended to control steady-state concentrations in veterinary patients with plasma sampling and analysis of trough levels before the next drug administration. Standardized drug level monitoring schemes are in general highly recommended. In a recent study comparing the effect of timing of blood collection on serum phenobarbital concentrations in dogs, no difference was evident between trough, 3-hour and 6-hour concentrations indicating that timing of blood sampling is not as important when phenobarbital is administered twice daily [49, 50]. However, timing of sampling is likely to be of relevance, when antiepileptic drugs marketed for veterinary patients have failed to achieve seizure control resulting in the use of drugs developed and marketed for human patients. The pharmacokinetic features of respective antiepileptic drugs are often suboptimal for dogs and cats, and have often not been studied in detail in veterinary patients. Thus, the choice of adequate administration intervals requires careful consideration and control by determining trough levels. Determining trough concentrations is also of particular interest, if seizures predominantly occur during the night. Moreover, it needs to be considered that small changes in plasma concentrations might adversely affect outcome in an individual patients, while no statistical effect might be observed in a larger study population.

This however also requires valid knowledge about the therapeutic plasma concentration range in dogs and cats, which is not available for all antiepileptic drugs, which have been used in canine and feline patients. Moreover, putative drug interactions need to be considered with polytherapeutic regimens. Muñana et al [51] have recently reported that concurrent administration of phenobarbital alone or together with bromide significantly alters the disposition of the antiepileptic drug levetiracetam compared to co-administration of bromide alone. In line with previous findings from healthy dogs [52], the findings pointed to the fact that phenobarbital lowers maximum plasma concentrations reached and accelerates the clearance of levetiracetam in epileptic dogs [51]. A similar interaction with phenobarbital has been shown for zonisamide [53, 54].

As pointed out above, seizure freedom is the primary goal in the therapeutic management of epilepsy patients. An intense discussion has dealt with the minimum duration of a therapeutic trial allowing conclusions about seizure-freedom in the course of an intervention trial. Being considered seizure-free has major implications for a human epilepsy patient for instance affecting the allowance to drive or to work in specific environments. In veterinary medicine the main question is whether the duration of a trial has been long enough to be informative, so that one can decide about continuing with another intervention trial in the case of therapeutic failure. Moreover, trial duration also has significant implications for the design of clinical studies, which require specific ethical as well as trial validity considerations as further discussed below.

A task force established by the ILAE has proposed that a patient should be considered seizure-free in response to a new intervention once no seizure occurred “during a phase of at least three times the duration of their longest pre-intervention interseizure interval in the preceding 12 months or during 12 months, whichever is longer” [1]. Evaluation during a time span of at least three times the duration of their longest preintervention interseizure interval has been reported to result in a 95 % certainty that the patient’s seizure frequency has at very least been decreased [1]. However, it has also been emphasized that this certainty is only reached in patients with a high seizure frequency. The ILAE task force proposal is based on the statistical principle referred to as the ‘Rule of Three’, which dealt with the issue to calculate confidence intervals for zero events [55, 56]. The minimum duration of seizure freedom for 12 months has been added by the Task Force in order to obtain information, if a clinically relevant sustained effect occurred [1]. If seizure freedom of at least three times the longest preintervention seizure interval has been reached but for less than 12 months, the outcome regarding seizure control is considered “undetermined” until seizure freedom lasts for at least 12 months [1]. More recently, Westover et al [57] have stated that the “Rule of Three” as an operational definition of seizure freedom might be reasonable in many cases, but that in other common cases a longer waiting time might be necessary. The authors suggested a revised criterion for seizure freedom which they termed the ‘Rule of Three-to-Six’ [57]. This suggestion considers the pre-intervention probability for therapeutic success, which for instance can be significantly reduced in patients with a history of multiple failed therapeutic trials. In veterinary medicine valid data is lacking so that it is not possible to reliably conclude about pre-intervention probability. Thus, it is recommended to consider the ILAE Task Force proposal as a basis for seizure outcome classification in veterinary patients. However, the statistical limitations, which are most pronounced in patients with low seizure frequencies, need to be considered when drawing conclusions.

In this context, it is important to note that the development of tolerance has been reported in canine patients during the course of a chronic antiepileptic drug treatment regime. Thereby, one needs to distinguish between metabolic tolerance related to accelerated drug metabolism and elimination rates and functional tolerance related to alterations in drug targets sites. Whereas metabolic tolerance might be overcome by adjustment of dosing or administration intervals, this might not be possible with functional tolerance.

The phenomenon of tolerance also referred to as the ‘honeymoon effect’ can result in relapse after prolonged periods of a pharmacological treatment. Tolerance development has for instance been suggested by studies with zonisamide or levetiracetam add-on regimens [58, 59]. However, in these studies the relapse or impairment of seizure control occurred within 2 and 8 months following initiation of the new therapeutic regime [58, 59]. Thus, the fact that the seizure-free period should last at least 12 months according to the ILAE Task Force proposal should account for most of the cases with tolerance development in canine patients rather avoiding a bias of the ‘honeymoon effect’ on seizure outcome conclusion. However, it is also emphasized that relapse is possible later on, and that a continued follow up of seizures during subsequent years is crucial in order to conclude about clinically relevant long-term success. In this context, it is also important to consider that seizure reoccurrence during therapy might also reflect ‘regression to the mean’ as patients often enter trials, when seizure frequency is high, and for the first few months seizure frequency might just be reduced due to the natural course of individual seizure frequency fluctuation. Please note that the definition of short term and long term therapeutic success is discussed in detail below (see subchapter on *Outcome criteria for clinical studies*).

Several issues can result in the owners’ and practitioners’ decision for discontinuation of a specific intervention. In these cases, it is of utmost importance to document the reasons for discontinuation in the patient’s files indicating whether tolerability issues, lack of efficacy, lack of compliance, financial considerations or other reasons resulted in the decision. Respective information will be of relevance for future therapeutic management decisions throughout the patient’s life, and will be of particular significance if the patient is enrolled in future clinical studies.

## Assessment of outcome: implications for clinical studies

### Ethical and general aspects

There is a great interest of owners of epileptic dogs to participate in trials of new antiepileptic drugs and regimens. This interest is driven by failures of available antiepileptic drugs in a proportion of epileptic dogs and concerns about possible side effects of antiepileptic drug treatment [12, 60–62]*.* There is general consensus that an informative clinical trial of antiepileptic drugs should be conducted in a controlled, blinded and randomized manner in order to achieve a high level of evidence [63] and to adjust for placebo effects which may average up to 30 % [64], and which have been explained by natural fluctuations in seizure frequency; but underreporting of seizures towards the end of the trial or improper patient selection may also increase clinical outcome variability [65, 66].

This raises several ethical issues which are of relevance to epileptic dogs and their owners. In particular, there are concerns that participation in placebo-controlled clinical studies may withhold the chance for successful treatment by the next individual therapeutic trial, either due to the use of placebo or because of the requirements to stay on an ineffective drug regime for prolonged time periods in order to complete the study with a sufficient number of subjects and to assess monthly seizure frequency within a fixed treatment period.

These issues may be approached by the use of direct comparison head-to-head trials and application of outcome parameters which allow individual study end points. Comparative head-to-head trials compare the effectiveness of the drug under investigation against another drug, usually a licensed drug with proven effectiveness against placebo considered the gold standard for the specific indication (active control; e. g. phenobarbital) [67]. This approach should provide each study participant with a highly effective antiepileptic drug, but has the draw-back that the differences between the interventional group and the control group are smaller than if compared to placebo and that higher numbers of participants are required for demonstration of smaller effects. Assessment of outcome in clinical studies requires definition of clearly defined primary outcome measures. The primary outcome measure in AED trials is efficacy defined by the drug’s influence on seizure occurrence, but tolerability, quality of life, compliance and retention rates should also be assessed in informative clinical trials [65].

The use of individual outcome parameters in clinical trials which define individual study end points will be possible if clinical studies aim at seizure freedom [1]*.* This has been suggested in human medicine for a long time (e. g. time to first seizure, time to n^th^ seizure, or individual patient-centered outcome criteria including tolerability issues), but clinical studies utilizing these outcome parameters are rarely found and validation for veterinary patients would be required [65, 66, 68].

A more detailed discussion about general aspects regarding the design of clinical studies is beyond the scope of this paper. In this consensus statement we will focus on outcome parameters, and will just shortly introduce the different types of clinical studies because study design and inclusion criteria will affect assessment of outcome.

### Types of clinical studies

Clinical studies of AED therapy should clearly describe the study goal and the study population in focus. The two different types of AED treatment trials are: (1) Evaluation of AED monotherapy, or (2) evaluation of adjunctive AED add-on therapy. The study populations in focus for the two study designs differ by chronicity and likelihood of a positive outcome: Evaluation of AED monotherapy focusses on patients with new onset epilepsy, while the study population for evaluation of AED add-on therapy is more likely to be composed of epilepsy patients with a history of recurrent seizures for prolonged time periods up to several years and proven refractoriness to several AEDs. Controlled randomized clinical studies with inclusion of control groups provide higher levels of evidence and are preferred to uncontrolled open label pilot studies of antiepileptic drug efficacy. In the latter each patient serves as its own control and seizure frequency during the intervention period is compared to a comparable baseline period. Uncontrolled open label studies cannot differentiate between drug effects, natural disease fluctuations (placebo response) and systemic influences e. g. intensified patient care during the treatment period which might affect seizure frequency. Still, open label pilot studies allow preliminary conclusions as to the potential efficacy of the drug under investigation, and can provide baseline statistical data for calculation of the necessary group sizes to conduct meaningful controlled clinical studies with adequate statistical power. The major reason why controlled randomized clinical studies fail to show an existing effect is inadequate statistical power, caused by high drop-out rates, insufficient patient numbers in the intervention and control group to show an effect of a given size, or high placebo responses [48, 64]. Patient selection contributes to the variance within and between treatment groups and will be further outlined below as part of the inclusion criteria. Clinical studies in epilepsy also differ significantly by the types of controls, which are used in the respective studies. These should be clearly described to facilitate interpretation of the results. Four different types of controls are distinguished: (1) placebo, which should have a similar appearance as the drug, (2) pseudoplacebo, meaning that the active drug is provided to the control group in a low dose which may not be effective (3) active control (positive control, treatment with an effective drug provided to the control group, head-to-head trial) (4) pseudocontrol (control group without any treatment; also termed negative control) [69]. Often, for ethical reasons the only choice for a trial in patients with new onset epilepsy can be an active control trial [67]. In line with this concept, Boothe et al [70] have performed a head-to-head trial comparing the efficacy and tolerability of phenobarbital and bromide as an initial monotherapy. During the development of imepitoin as a new AED for canine epilepsy, several trial types have been used in epileptic dogs [19]: (1) an open (non-controlled) trial, comparing imepitoin with phenobarbital and primidone in newly diagnosed dogs with epilepsy; (2) an open (non-controlled) trial, comparing add-on with imepitoin with add-on with potassium bromide in dogs resistant to treatment with phenobarbital and primidone; (3) a randomized controlled trial with imepitoin vs. pseudoplacebo (low dose of imepitoin); (4) a randomized controlled trial with imepitoin vs. primidone; and (5) a randomized controlled trial with imepitoin vs. phenobarbital. The last trial type was used in a pivotal field trial for approval of imepitoin by the European Medicines Agency [20]. A randomized placebo-controlled trial for approval in the U.S. is in progress. As an alternative the use of historic controls is intensely discussed in human medicine [71, 72]. However, due to alterations in study populations, and placebo response rates over time and due to a pronounced impact of study sites on outcome, the use of historic controls also faces major issues. In veterinary medicine, the paucity of well-controlled studies represents another limitation. In general, patients should be assigned to the intervention and control groups in a blinded and randomized manner to avoid any bias in patient selection. However, stratification of treatment groups for disease severity and further parameters (e.g. diagnosis, seizure type, appearance of cluster seizures, age of onset, duration of seizures prior to treatment, breed) may be warranted. Moreover, strict inclusion and exclusion criteria need to be applied considering respective parameters and clearly defining the study population (Table 2).

**Table 2:** Important inclusion criteria which may affect outcome

Criteria for the diagnosis of epilepsy	How exclusion of other episodic events (paroxysmal dyskinesias, tremors, episodic collapse etc.) is achieved, which are likely not to respond to interventions with AEDs
Criteria to restrict the study population under investigation to specific patient groups, breed-specific epilepsies, specific etiologies	E. g. if restriction to patients with idiopathic epilepsy, implies to define specific measures and examinations undertaken to exclude other causes of epilepsy that are known to influence outcome in a significant manner. These criteria should follow the requirements for the diagnosis of idiopathic epilepsy as defined in a separate consensus statement.
	Description of the specific pharmacoresistance pattern of the study population under investigation e. g. resistance to phenobarbital, potassium bromide, imepitoin, levetiracetam etc. Definition of pharmacoresistance should follow previous consensus in this paper, which may include definition of minimum serum concentrations, requirements for measurement of trough levels and definition of steady state periods as deemed necessary based on mechanism of action for the specific drug for which pharmacoresistance is defined.
Criteria to restrict the study population to a specific disease stage	Criteria to restrict the study population under investigation either to
	• trials of AEDs in patients with new onset epilepsy, or
	• trials of AEDs in patients with chronic refractory epilepsy
Criteria for pre-drug assessment in trials of AEDs in patients with chronic epilepsy	Definition of baseline data (e.g. written seizure diary, prospective, retrospective) and duration of baseline period for assessment of median pre-treatment seizure frequency, cluster seizure frequency or assessment of the longest seizure-free interval in the year preceding study inclusion
Criteria to restrict the study population to patients without severe systemic disease which will likely affect outcome	E. g. exclude severe preexisting hepatic, renal, endocrine disease

In this context it should be mentioned that the preferred type of study varies by the intention of the respective investigators e. g. regulatory ministries, drug companies, or clinicians treating the respective patients. The U.S. Food and Drug Administration (FDA) often requires statistical proof of superiority to a drug with known efficacy, European Medicines Agency (EMEA) requires proof of noninferiority.

### Outcome criteria for clinical studies

In human patients huge differences exist between epilepsies of different etiologies and seizure types regarding responsiveness to different interventions, while only limited data are available in veterinary neurology with regard to different types of epilepsies or breed-specific epilepsy syndromes. It is generally agreed that a gain in knowledge can only be obtained by applying stringent inclusion criteria and defined endpoints, which define the patient groups under investigation and, furthermore, set the base for large multicenter studies with adequate statistical power. Inclusion criteria and outcome parameters must be identical for the intervention and control group, in order to avoid any bias which may influence outcome assessment. Important inclusion criteria which may influence outcome of clinical studies are outlined shortly in Table 2, while the further discussion focuses on the specific outcome parameters.

Regarding outcome criteria it is recommended to consider all categories discussed above for individual patients, and to apply standardized evaluation tools for assessment. Thus, the outcome assessment should not only consider the impact on seizures (efficacy), but also a detailed evaluation of adverse effects (tolerability) and of the impact of the intervention on behavioral comorbidities, and on quality of life of the patient and caretaker (Table 3). With regard to tolerability detailed data on reasons for study drop-out should be provided for each patient that exits prematurely. Furthermore retention rate is a clinical relevant parameter which reflects the percentage of patients adhering to the drug after prolonged treatment periods and thus is considered a useful parameter for combined assessment of efficacy, tolerability and even quality of life. Regarding the intervention’s impact on seizures as many data should be collected as possible. These should include total number of seizures, seizure days allowing calculation of median seizure frequency and seizure day frequency and seizure free intervals (days). Additional parameters that assess severity (occurrence of clusters and average number of seizure per cluster, status epilepticus, focal seizures vs. generalized seizures, severity and duration of post ictal signs) should be included. This would allow assessment of outcome in concordance with current recommendations in human medicine. Conventional primary outcome parameters in humans are seizure free rate, median seizure frequency, and responder rate, whereby a drug responder is defined by a > 50 % reduction in seizures compared to baseline. However, this is generally considered a very weak endpoint, also reached by placebo in many patients, so that many clinical studies prefer at least 75 % reduction in seizure frequency. It should be noted that responder rate may not be a clinically meaningful outcome parameter, while assessment of the seizure free rate (percentage) is a hard outcome parameter which is independent from baseline data and is clinically relevant. Current ILAE guidelines request a ≥ 20 % absolute difference in between treatment groups for ascertainment of a clinically relevant positive outcome [73, 74]. It remains debatable whether a 20 % difference in outcome constitutes a clinically relevant difference in veterinary patients. A summary of outcome criteria which highlight different aspects of the disease and are currently discussed in human medicine is provided in Table 2. Study protocols and assessment schemes should also collect information about putative seizure precipitating events or factors (e.g. owner leaving, excessive activity, transfer to kennel).

**Table 3:** Summary of primary outcome endpoints which are applicable to clinical studies and highlight different aspects of outcome; modified from [65, 66, 73]

Outcome parameters	
Efficacy	
Conventional endpoints	fixed treatment period
Seizure free rate (percentage seizure freedom)^a^	no baseline data required
Short-term	24 weeks
Long-term	48 weeks – 3 years
Median seizure frequency reduction	baseline data required
Responder rate (percentage responders)	baseline data required
(≥50 % reduction median seizure frequency often not clinically relevant)	
Individual endpoints	
Time to first seizure	based on interseizure interval
Time to second seizure	
Time to n-th seizure	
Pre-defined patient-centered outcome criteria	individually assessed
Tolerability	
Adverse events	to be assessed, also assess number and reasons for drop-outs
Quality of life	
Patient’s QOL score	validated scores needed
Owner’s QOL score	control groups important
Retention rate	applicable to long-term studies

^a^ Need to specify reliability of assessment: (A) freedom of generalized seizures only or (B) freedom of generalized and focal seizures

A consensus was reached within the group that defined individual study end points based on individual pretreatment seizure frequency are preferred and that respective study designs should be further developed and validated in accordance to suggestions for AED trials in human patients [1]. Preferred endpoint was the definition of short term success as seizure freedom for a time-span exceeding three times the longest interseizure interval (days) in the year preceding the study and for a minimum of three months (time to 1^st^ seizure) [1, 65, 66]. Thus, if seizure freedom is not achieved time to the 2^nd^ or n^th^ seizure was considered as an alternative outcome parameter for add-on trials in patients with chronic refractory epilepsy [1, 65]. In this setting, any patient with continued seizures following a titration phase will be classified as treatment failure and allowed to exit the study. Consequently, patients with complete freedom of seizures or extension of the interseizure interval to three times the longest interseizure interval and a minimum of three months will be considered treatment success and treatment should thereafter be continued to assess the seizure free rate e. g. the percentage of patients with short-term or long-term freedom of seizures [67].

The use of seizure freedom as a primary outcome parameter follows current ILAE recommendations and has been successfully applied as primary outcome parameter in one veterinary study focusing on new onset epilepsy (outcome described as percentage seizure freedom, short term) [70]. With this approach differences in seizure frequency, seizure days, seizure severity, clusters or status epilepticus during a fixed time period between groups should be considered secondary outcome parameters in clinical studies which can define and describe partial treatment success in patients with chronic epilepsy participating in add-on trials of AEDs in which seizure freedom may be difficult to achieve.

Open questions remain as to the definition of short term or long term treatment success and whether seizure freedom can be a realistic goal in chronic epileptic patients with AED drug polytherapy. Consensus exists that the minimum duration of 24 weeks for studies in human patients only assesses short term response to AEDs, is subject to the so-called honeymoon effect, and does not adequately predict long-term outcome after 1 year, 2 years or 5 years of treatment. Thus, follow-up of patients for up to one year or even longer is warranted. Besides seizure frequency, dogs’ QoL, owners’ QoL, adverse effects affecting tolerability, retention rate of AED, survival rates and number and costs of veterinary visits are other outcome parameters which may be specifically applicable to long-term clinical studies in veterinary patients due to the shortened life span of dogs and cats compared to humans and the specific human-animal-bond, which is affected by the disease. Open questions remain also to the reliable assessment of focal seizures in clinical studies in veterinary patients. Can these be reliably counted and assessed in clinical studies in veterinary patients without use of invasive EEG based recording tools? Should improvement in generalized but not in focal seizures be rated as a positive outcome e. g. partial treatment success? These thoughts are especially important if seizure freedom is applied as the primary outcome parameter.

Further important points to be discussed are whether stratification of treatment and control groups for appearance of cluster seizures, breed and age of onset should be attempted. Specifically the frequent appearance of cluster seizure events appears to characterize a difficult to treat subpopulation in veterinary patients with idiopathic epilepsy [12, 62, 75]. Differences between certain dog breeds appear to exist in regard to the natural course of the epilepsy, while the impact of other factors (e. g. previous head trauma) on outcome needs yet to be defined. However, a more detailed discussion about general aspects regarding the design of clinical studies and the influence of study design on outcome assessment is beyond the scope of this paper and will be provided in a separate publication.

## Abbreviations

SUDEP: Sudden unexpected death in epilepsy
ILAE: International League against Epilepsy
QoL: Quality of life
AED: Antiepileptic drug
FDA: Food and drug administration
